# Supplementary material for: Metabolic syndrome predictive modelling in Bangladesh applying machine learning approach
Source: PLoS One. 2024 Sep 5;19(9):e0309869. doi: 10.1371/journal.pone.0309869 (PMC11376561; doi:10.1371/journal.pone.0309869)
Supplement: Attachment — Submitted filename: Response to Reviewer.pdf [file pone.0309869.s002.pdf]

Manuscript Number: PONE-D-24-05709

Author's response to the reviewer's comments

Based on the Reviewer's comments Author respond are the followings

Reviewer 1:

Reviewer comments	Responses
The abstract should clearly state the main objectives, methodology, key results, conclusions, and limitations of the study. Currently it only summarizes the background.	We appreciate your feedback. The primary goals, methodology, important findings, conclusions, and limitations of the study have all been spelled out in detail in the abstract.
Include the key risk factors identified, prevalence of MetS found, and main ML model utilized in the results.	Thank you for your recommendations. The major machine learning model used in the results, the prevalence of MetS, and the key risk variables revealed have all been presented in the "abstract".
Expand more on the rationale for identifying risk factors and predicting MetS prevalence in the Bangladeshi population specifically.	We appreciate your recommendations. The reasoning behind determining risk variables and forecasting the incidence of MetS in the Bangladeshi population in particular is covered in greater detail in the "Introduction section" of our publication.
after 'and working in irregular shifts [7].'discuss the role in particular of obstructive sleep apnea. cite doi:10.3390/life13030702.	Thanks for your suggestions. According to this research doi:10.3390/life13030702, we have addressed the role of obstructive sleep apnea specifically in the "Introduction section."
Provide more background on MetS diagnostic criteria and associated health risks to establish significance.	We appreciate your recommendations. To establish significance, we have included further background information on MetS diagnostic criteria and related health hazards in the "Introduction section" of our publication.
Describe the National STEPS Survey sampling methodology, data collection procedures, and variables obtained in more detail.	Thank you for your recommendations. More information on the National STEPS Survey sampling methodology, data collection techniques, and variables gathered may be found in the section titled "Data Source in the Methodology section."
Explain how MetS was defined - which diagnostic criteria used	Thanks for your suggestionsThe definition of MetS and the diagnostic criteria that were

	applied are covered in the section on "diagnostic criteria in the Methodology section."
Specify why certain variables were categorized and encoding approaches for ML models	We have explained why specific variables were categorized and encoding methodologies for ML models in the section "Variable importance and key risk factors of metabolic syndrome in the Results and analysis section."
State specific ML models, parameters, evaluation metrics, and statistical analysis done	Thanks for your suggestions. Certain machine learning models, parameters, assessment metrics, and statistical analysis have been specified in the "Results and analysis" portion of Tables 2, 3, and 4.
Focus this section only on key results - prevalence, risk factors, model performance. Remove general discussion	We appreciate your recommendations. We only included the most important findings—prevalence, risk variables, and model performance—in the "Results and analysis" section.
Include quantitative performance metrics for each ML model - precision, recall, ROC, etc.	Thank you for your tremendous recommendations. We incorporated numerical performance indicators, such as precision, recall, ROC, and so on, for every machine learning model in our manuscript's "Results and analysis section in Table 4."
Limitations should note small subset of survey data used, exclusion of potentially relevant variables like lipids. cite doi:10.1016/j.compbiomed	We appreciate your recommendations. We have mentioned in the "Limitations Section" that a tiny subset of survey data was utilized, that potentially relevant factors like lipids were excluded, and that we cited this article doi:10.1016/j.compbiomed.
Discussion of inflammation markers and future studies seems speculative without any results presented. Would remove.	We appreciate your insightful recommendations. The part about future research and signs of inflammation has been removed.
Avoid overstating conclusions on predictive capabilities of models until externally validated	Thanks for your valuable suggestions. This has been deleted from the "Conclusion section."

Reviewer #2

Reviewer comments	Responses
It's crucial to assess how well the models generalize to unseen data. Cross-validation techniques and external validation on datasets from different populations could enhance the robustness of the findings.	Your insightful recommendations are appreciated. After examining datasets from various populations, we have conducted cross-validation and external validation on our models and found that they perform well.
The study could benefit from a direct comparison with existing predictive models for MetS to highlight its contributions or improvements	We appreciate your insightful recommendations. There are no current MetS prediction models available that we can compare our model to from the standpoint of the Bangladeshi population. Our models showed marginally less accuracy when we compared them to the state-of-the-art models from China and Mexico.
While the study employs feature selection techniques, further exploration into dimensionality reduction methods could improve model performance and interpretability.	Thanks for your valuable suggestions. Although we have looked into dimensionality reduction techniques, our model's performance remains unchanged.
The exclusion of certain variables deemed insignificant could potentially overlook complex interactions between features that contribute to MetS. A more detailed analysis or justification for the exclusion of these variables might provide deeper insights.	Table 5 in the "Results and analysis section" illustrates how these variables lower the models' overall performance. Because of this, we have decided to eliminate these factors from our study because they don't really matter for our findings. We think more research is necessary to ignore the intricate relationships between the characteristics that cause MetS.
The practical applicability of the models in clinical settings is not extensively discussed. Future work could focus on how these models can be integrated into healthcare systems for early detection and intervention strategies	We appreciate your recommendations. The "Discussion and recommendations section" of our publication contains a brief discussion of the models' usefulness in clinical settings. To further understand how machine learning models might be incorporated into Bangladeshi healthcare systems for early detection and intervention measures, we think more research is needed.
Mention briefly deep learning methods article and add in the reference. For example: "An Adaptive Ensemble Deep Learning	Thanks for your important suggestions. In the "Related work section" of our manuscript, we have included a brief reference of an article

Framework for Reliable Detection of Pandemic Patients", "Deep Learning Network Selection and Optimized Information Fusion for Enhanced COVID-19 Detection". Deep learning could offer insight into the future research direction.	on deep learning that may provide some light on potential future study directions.

Reviewer #3

**Reviewer comments**	**Responses**
This basic and general information is not necessary. The authors should write procedures the authors performed in this study, but no information is provided in this manuscript, which is not appropriate. The authors should provide more detailed information of procedures, such as R and its version, packages, parameters used in analysis, etc.	Thanks for your suggestions. We removed the general discussion as possible. We included the author’s contribution in this manuscript. We provided detailed information of procedures. We used SPSS Python instead of R. We included its version, libraries and parameter used in analysis.
